# Supplementary material for: Metasurface-assisted massive backscatter wireless communication with commodity Wi-Fi signals
Source: Nat Commun. 2020 Aug 6;11:3926. doi: 10.1038/s41467-020-17808-y (PMC7413398; doi:10.1038/s41467-020-17808-y)
Supplement: Supplementary file 1 — Supplementary Information [file 41467_2020_17808_MOESM1_ESM.pdf]

Supplementary Information for

Metasurface-Assisted Massive Backscatter Wireless Communication with Commodity Wi-Fi Signals

Hanting Zhao[1+], Ya Shuang[1+], Menglin Wei[1], Tie Jun Cui[2*], Philipp del Hougne[3*], and Lianlin Li[1*]

[1] State Key Laboratory of Advanced Optical Communication Systems and Networks, Department of Electronics, Peking University, Beijing 100871, China

[2] State Key Laboratory of Millimeter Waves, Southeast University, Nanjing 210096, China

[3] Univ Rennes, CNRS, Institut d'Electronique et de Télécommunications de Rennes (IETR) - UMR 6164, 35000 Rennes, France

[+] These authors contributed equally to this work.

* Correspondance to tjcui@seu.edu.cn, philipp.delhougne@gmail.com, lianlin.li@pku.edu.cn.

Supplementary Note 1. The physical model of our MBWC technique

Here, we will elaborate on the physical model of the proposed MBWC. For simplicity, we consider the case of binary information quantization; however, the presented methods can be readily extended for more complicated cases. As shown in **Figure 1 in main text**, the proposed MBWC system is composed of several modules including a programmable metasurface, two or more coherent receivers, and an information decision module. When the programmable metasurface configured with the coding pattern $\mathcal{C}_0$ (or, $\mathcal{C}_1$) is illuminated by ambient stray wireless wave, two coherent receivers located at $\mathbf{r}_\mathrm{m}$ and $\mathbf{r}_\mathrm{s}$ are deployed to acquire the resultant echoes denoted by $y(\mathbf{r}_\mathrm{m}, \omega; \mathcal{C}_0)$ and $y(\mathbf{r}_\mathrm{s}, \omega; \mathcal{C}_0)$ (or $y(\mathbf{r}_\mathrm{m}, \omega; \mathcal{C}_1)$ and $y(\mathbf{r}_\mathrm{s}, \omega; \mathcal{C}_1)$). Put formally, the information retrieval of the MBWC can be formulated into a typical classification problem, i.e.,

$$I_\mathrm{out} = 1 \ , \quad \text{if } \frac{f\left(y(\mathbf{r}_\mathrm{m}, \omega; \mathcal{C}_1), y(\mathbf{r}_\mathrm{s}, \omega; \mathcal{C}_1)\right)}{f\left(y(\mathbf{r}_\mathrm{m}, \omega; \mathcal{C}_0), y(\mathbf{r}_\mathrm{s}, \omega; \mathcal{C}_0)\right)} \geq \eta;$$
$$= 0, \quad \text{else.} \qquad (1)$$

Herein, η is a decision threshold, $f(\cdot)$ is a real-valued prior-specified nonlinear function, and ω is the angular frequency of wireless signals. The retrieved information bit is "1" if $I_\mathrm{out} = 1$, corresponding to the case that the control coding pattern $\mathcal{C}_1$ is encoded into the metasurface. Similarly, the retrieved information bit is "0" when the coding pattern $\mathcal{C}_0$ is encoded into the metasurface.

In a nutshell, two critical issues are involved for the proposed MBWC. First, $\mathcal{C}_0$ and $\mathcal{C}_1$ need to be well optimized such that the decision rule (Supplementary Equation 1) can be implemented in a robust way. Second, it is crucial to choose a suitable information decoding scheme, i.e., to choose the nonlinear feature-extraction function $f(\cdot)$.

We consider the case that a single unknown wireless source is located somewhere, which represents a typical scenario of local wireless network, for instance, Wi-Fi. Specifically, for a given operational frequency channel, only one Wi-Fi router works at the same time within a relatively local environment. Here, we consider to realize the MBWC by using the programmable metasurface to manipulate the ambient stray wireless waves arising from the unknown wireless source. Without loss of generality, we assume that the unknown wireless source at $\mathbf{r}_1$ emits an unknown radio signal $\tilde{s}(\omega)$, as sketched in **Supplementary Figure 1a**. Then, the echoes received by two coherent receivers reads:

$$y(\mathbf{r}_\mathrm{m},\omega,\mathcal{C}) = \tilde{s}(\omega)G(\mathbf{r}_\mathrm{m},\mathbf{r}_1,\omega) + \tilde{s}(\omega)\textstyle\sum_n \sigma_n(\mathcal{C},\omega)G(\mathbf{r}_\mathrm{m},\mathbf{r}_n,\omega)G(\mathbf{r}_n,\mathbf{r}_1,\omega) \quad (2)$$

and

$$y(\mathbf{r}_\mathrm{s},\omega,\mathcal{C}) = \underbrace{\tilde{s}(\omega)G(\mathbf{r}_\mathrm{s},\mathbf{r}_1,\omega)}_{\text{direct arrival signal}} + \underbrace{\tilde{s}(\omega)\textstyle\sum_n \sigma_n(\mathcal{C},\omega)G(\mathbf{r}_\mathrm{s},\mathbf{r}_n,\omega)G(\mathbf{r}_n,\mathbf{r}_1,\omega)}_{\text{reflection signal}}, \quad (3)$$

respectively. Herein, $\sigma_n(\mathcal{C},\omega)$ represents the reflection coefficient of the nth meta-atom when the metasurface is configured with the coding pattern $\mathcal{C}$, and G denotes the Green's function of the surrounding environment. The Green's function formalism is very general and accounts for any type of propagation environment. For instance, multipath effects as found in complex environments with scattering effects and reverberation, are accounted for. For binary information quantization, $\mathcal{C}$ has two choices, i.e., $\mathcal{C}_0$ or $\mathcal{C}_1$. The first term in Supplementary Equations 2 and 3 characterizes the direct arrival from the unknown wireless source to the receivers, i.e. rays that did not encounter the programmable metasurface. The second term in Supplementary Equations 2 and 3 characterizes rays that interacted with the metasurface. Only the latter carry the information encoded by Alice into the metasurface configuration. For notational convenience, the argument ω has been deliberately suppressed below.

Now, we perform the mutual coherence of $y(\mathbf{r}_\mathrm{m},\mathcal{C})$ and $y(\mathbf{r}_\mathrm{s},\mathcal{C})$, and arrive at

$$\begin{aligned}
&\langle y(\mathbf{r}_\mathrm{m},\mathcal{C})y^*(\mathbf{r}_\mathrm{s},\mathcal{C})\rangle \\
&= \langle|\tilde{s}|^2\rangle G(\mathbf{r}_\mathrm{m},\mathbf{r}_1)\big(G(\mathbf{r}_\mathrm{s},\mathbf{r}_1)\big)^* \\
&+\langle|\tilde{s}|^2\rangle G(\mathbf{r}_\mathrm{m},\mathbf{r}_1)\big(\textstyle\sum_n \sigma_n(\mathcal{C})G(\mathbf{r}_\mathrm{s},\mathbf{r}_n)G(\mathbf{r}_n,\mathbf{r}_1)\big)^* \\
&+\langle|\tilde{s}|^2\rangle\big(\textstyle\sum_n \sigma_n(\mathcal{C})G(\mathbf{r}_\mathrm{m},\mathbf{r}_n)G(\mathbf{r}_n,\mathbf{r}_1)\big)\big(G(\mathbf{r}_\mathrm{s},\mathbf{r}_1)\big)^* \\
&+\langle|\tilde{s}|^2\rangle\big(\textstyle\sum_n \sigma_n(\mathcal{C})G(\mathbf{r}_\mathrm{m},\mathbf{r}_n)G(\mathbf{r}_n,\mathbf{r}_1)\big)\big(\textstyle\sum_n \sigma_n(\mathcal{C})G(\mathbf{r}_\mathrm{s},\mathbf{r}_n)G(\mathbf{r}_n,\mathbf{r}_1)\big)^* \quad (4)
\end{aligned}$$

Herein, the symbol $\langle\cdot\rangle$ denotes the statistical mean or ensemble average.

In order to facilitate the information retrieval or information demodulation in a robust way, we introduce an important assumption, i.e., <u>the slavery receiver at $\mathbf{r}_\mathrm{s}$ is "invisible" to the metasurface; meanwhile, the master receiver at $\mathbf{r}_\mathrm{m}$ is "visible" to the metasurface in an enhanced manner</u>. As a result, we have

$$|\textstyle\sum_n \sigma_n(\mathcal{C})G(\mathbf{r}_\mathrm{s},\mathbf{r}_n)G(\mathbf{r}_n,\mathbf{r}_1)| \ll |G(\mathbf{r}_\mathrm{s},\mathbf{r}_1)|$$

and

$$|\textstyle\sum_n \sigma_n(\mathcal{C})G(\mathbf{r}_\mathrm{s},\mathbf{r}_n)G(\mathbf{r}_n,\mathbf{r}_1)| \ll |\textstyle\sum_n \sigma_n(\mathcal{C})G(\mathbf{r}_\mathrm{m},\mathbf{r}_n)G(\mathbf{r}_n,\mathbf{r}_1)|$$

Then, Supplementary Equation 4 becomes,

$$\langle y(\mathbf{r}_\mathrm{m},\mathcal{C})y^*(\mathbf{r}_\mathrm{s},\mathcal{C})\rangle \approx \langle|\tilde{s}|^2\rangle G(\mathbf{r}_\mathrm{m},\mathbf{r}_1)\big(G(\mathbf{r}_\mathrm{s},\mathbf{r}_1)\big)^*$$

$$+\langle|\tilde{s}|^2\rangle\left(\sum_n \sigma_n(\mathcal{C})G(\mathbf{r}_{\mathrm{m}},\mathbf{r}_n)G(\mathbf{r}_n,\mathbf{r}_1)\right)\left(G(\mathbf{r}_{\mathrm{s}},\mathbf{r}_1)\right)^* \quad (5)$$

Apparently, **Supplementary Equation 5 can be treated as the demodulation procedure for the proposed MBWC**, as explained in main text. Now, two interesting observations can be made from Supplementary Equation 5. First, the first term of Supplementary Equation 5 is independent of the coding pattern $\mathcal{C}$ of the metasurface. Therefore, it can be calibrated out by using a simple averaging smooth or background removal operation for the time-varying control coding patterns. Second, the ambient stray wireless signals behavior is statistically stationary, and thus $\langle|s(\omega)|^2\rangle$ can be treated as a constant factor independent of the information encoded into the metasurface configuration. These results, due to the generality of the Green's function formalism, are not restricted to a free space propagation environment. They are equally valid in propagation environments with multipath effects. Indeed, focusing a speckle-like ambient field with a programmable metasurface is also possible in reverberant environments, as demonstrated in Ref.[1].

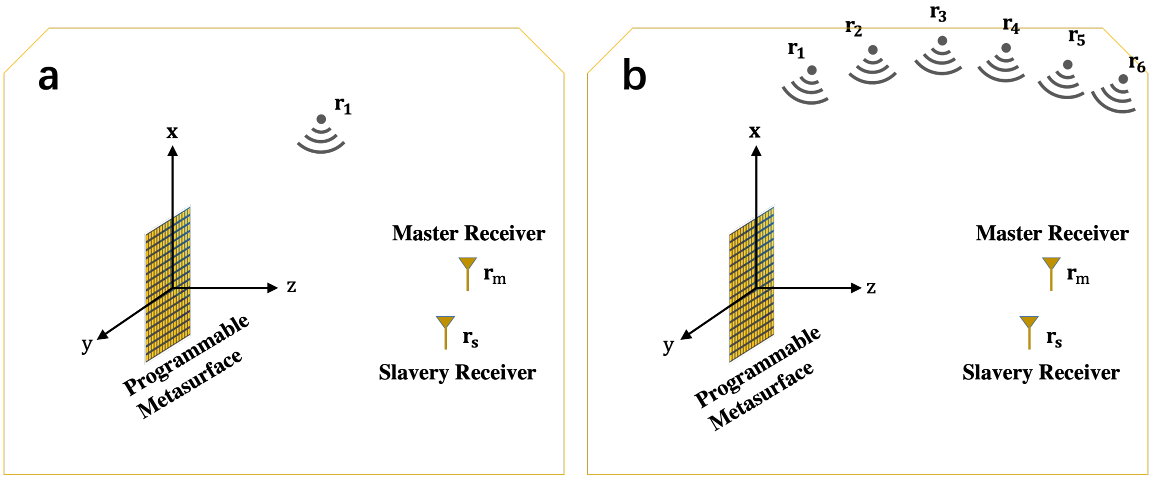

Supplementary Figure 1. Sketched maps for deriving Supplementary Equation 5 (left) and Supplementary Equation 13 (right). Left is for the case of a single unknown wireless source. Right is for the case of multiple unknown wireless sources.

Here, we list three more remarks on Supplementary Equation 5 as following:

Remark 1.

The "(in)visibility" can be readily achieved by controlling the radiation beam of the metasurface. In particular, the coding patterns, i.e., $\mathcal{C}_0$ or $\mathcal{C}_1$, can be optimized such that the radiation beam of the metasurface can be focused towards the local spot around the

master receiver; in contrast, it has remarkably lower energy around the slavery receiver. In this way, the master receiver is designed for acquiring the stray wireless signal carrying the digital information encoded into the metasurface, while the slavery receiver is only for sampling the wireless signal directly from the non-cooperative wireless source. As such, **the passive "carrier" signal can be well reallocated to the Alice and Bobs in a wireless and controllable way**.

Remark 2.

In order to achieve the robust information retrieval (i.e., information decoding), it is required that $\langle y(\mathbf{r}_{\mathrm{m}},\mathcal{C}_0)y^*(\mathbf{r}_{\mathrm{s}},\mathcal{C}_0)\rangle$ and $\langle y(\mathbf{r}_{\mathrm{m}},\mathcal{C}_1)y^*(\mathbf{r}_{\mathrm{s}},\mathcal{C}_1)\rangle$ are as distinguishable as possible in terms of a chosen metric, which can be realized by optimizing the control coding patterns of $\mathcal{C}_0$ and $\mathcal{C}_1$. For instance, we can maximize the Euclidean distance between the two:

$$|\langle y(\mathbf{r}_{\mathrm{m}},\mathcal{C}_0)y^*(\mathbf{r}_{\mathrm{s}},\mathcal{C}_0)\rangle-\langle y(\mathbf{r}_{\mathrm{m}},\mathcal{C}_1)y^*(\mathbf{r}_{\mathrm{s}},\mathcal{C}_1)\rangle|$$
$$=\langle|\tilde{s}|^2\rangle\left|\left(\sum_n\left(\sigma_n(\mathcal{C}_0)-\sigma_n(\mathcal{C}_1)\right)G(\mathbf{r}_{\mathrm{m}},\mathbf{r}_n)G(\mathbf{r}_n,\mathbf{r}_1)\right)G^*(\mathbf{r}_{\mathrm{s}},\mathbf{r}_1)\right| \qquad (6)$$

Supplementary Equation 6 shows that the optimal choice of the coding patterns for the binary MBWC modulation can be achieved by setting $\sigma_n(\mathcal{C}_1)=-\sigma_n(\mathcal{C}_0)$.

Remark 3.

Here, we consider the scenario of a set of statistically i.i.d. non-cooperative wireless sources $\{N_i(\omega), i=1,2,\dots\}$ which are spatially distributed outside the communication region Ω, as shown in **Supplementary Figure 1b**. The ith wireless source is located at $\mathbf{r}_i$. Similar to above, the wireless signals acquired by two receivers at $\mathbf{r}_{\mathrm{m}}$ and $\mathbf{r}_{\mathrm{s}}$ read:

$$y(\mathbf{r}_{\mathrm{m}},\omega;\mathcal{C})=\sum_i N_i(\omega)[G(\mathbf{r}_{\mathrm{m}},\mathbf{r}_i,\omega)+\sum_n\sigma_n(\mathcal{C},\omega)G(\mathbf{r}_{\mathrm{m}},\mathbf{r}_n,\omega)G(\mathbf{r}_n,\mathbf{r}_i,\omega)] \qquad (7)$$

and

$$y(\mathbf{r}_{\mathrm{s}},\omega;\mathcal{C})=\sum_i N_i(\omega)[G(\mathbf{r}_{\mathrm{s}},\mathbf{r}_i,\omega)+\sum_n\sigma_n(\mathcal{C},\omega)G(\mathbf{r}_{\mathrm{s}},\mathbf{r}_n,\omega)G(\mathbf{r}_n,\mathbf{r}_i,\omega)], \qquad (8)$$

respectively. Here, we still consider the case that the master receiver at $\mathbf{r}_{\mathrm{m}}$ is "visible" to the metasurface; however, the slavery receiver at $\mathbf{r}_{\mathrm{s}}$ is "invisible" to the metasurface. As a result, Supplementary Equations 7 and 8 become:

$$y(\mathbf{r}_{\mathrm{m}},\omega;\mathcal{C})=\sum_i N_i(\omega)[G(\mathbf{r}_{\mathrm{m}},\mathbf{r}_i,\omega)+\sum_n\sigma_n(\mathcal{C},\omega)G(\mathbf{r}_{\mathrm{m}},\mathbf{r}_n,\omega)G(\mathbf{r}_n,\mathbf{r}_i,\omega)] \qquad (9)$$

and

$$y(\mathbf{r}_\mathrm{s}, \omega; \mathcal{C}) = \sum_i N_i(\omega) G(\mathbf{r}_\mathrm{s}, \mathbf{r}_i, \omega), \quad (10)$$

Then, the coherence function of the signals $y(\mathbf{r}_\mathrm{m}, \omega; \mathcal{C})$and $y(\mathbf{r}_\mathrm{s}, \omega; \mathcal{C})$ reads:

$$\begin{aligned}
&\langle y(\mathbf{r}_\mathrm{m}, \mathcal{C}) y^*(\mathbf{r}_\mathrm{s}, \mathcal{C})\rangle \\
&= \sum_j \sum_i \langle N_i(\omega) N_j^*(\omega)\rangle [G(\mathbf{r}_\mathrm{m}, \mathbf{r}_i) + \sum_n \sigma_n(\mathcal{C}) G(\mathbf{r}_\mathrm{m}, \mathbf{r}_n) G(\mathbf{r}_n, \mathbf{r}_i)] G^*(\mathbf{r}_\mathrm{s}, \mathbf{r}_j) \\
&= C(\omega) \sum_i [G(\mathbf{r}_\mathrm{m}, \mathbf{r}_i) + \sum_n \sigma_n(\mathcal{C}) G(\mathbf{r}_\mathrm{m}, \mathbf{r}_n) G(\mathbf{r}_n, \mathbf{r}_i)]\, G^*(\mathbf{r}_\mathrm{s}, \mathbf{r}_i)
\end{aligned} \quad (11)$$

In Supplementary Equation 10, we have explored the fact that $\langle N_i(\omega) N_j^*(\omega)\rangle = C(\omega)\delta(i-j)$, where $C(\omega) = \langle |N_i(\omega)|^2\rangle$. Here, we consider an extreme case that the number of *i.i.d.* wireless sources tends to be infinity. In this case, we can arrive at the following conclusion in light of the well-known time-reversal theory:

$$\sum_j G(\mathbf{r}_\mathrm{m}, \mathbf{r}_j) G^*(\mathbf{r}_\mathrm{n}, \mathbf{r}_j) \approx h(\mathbf{r}_\mathrm{m} - \mathbf{r}_n) \quad (12)$$

where h is a shift-invariant kernel function. Using Supplementary Equation 12 in Supplementary Equation 11 then leads to:

$$\langle y(\mathbf{r}_\mathrm{m}, \mathcal{C}) y^*(\mathbf{r}_\mathrm{s}, \mathcal{C})\rangle \propto C(\omega)[h(\mathbf{r}_\mathrm{m} - \mathbf{r}_\mathrm{s}) + \sum_n \sigma_n(\mathcal{C}) G(\mathbf{r}_\mathrm{m}, \mathbf{r}_n) h(\mathbf{r}_n - \mathbf{r}_\mathrm{s})] \quad (13)$$

Upon comparing Supplementary Equation 13 with Supplementary Equation 5, the aforementioned conclusions can be drawn again.

Supplementary Note 2. Three representative (de)modulation schemes for the MBWC

We consider three MBWC (de)modulation schemes. For convenience, we introduce the notation of $\widehat{H}_{\mathrm{s}\to\mathrm{meta}\to\mathrm{mr}}(\mathbf{r}_\mathrm{m}, \omega; \mathcal{C}) = \sum_n \sigma_n(\mathcal{C}_1) G(\mathbf{r}_\mathrm{m}, \mathbf{r}_n) G(\mathbf{r}_n, \mathbf{r}_1)$.

- <u>Binary Amplitude Shift Keying (BASK)</u>

$$\begin{aligned}
I_\mathrm{out} &= 1; \quad if \ \frac{|\widehat{H}_{\mathrm{s}\to\mathrm{meta}\to\mathrm{mr}}(\mathbf{r}_\mathrm{m}, \omega; \mathcal{C}_1)|}{|\widehat{H}_{\mathrm{s}\to\mathrm{meta}\to\mathrm{mr}}(\mathbf{r}_\mathrm{m}, \omega; \mathcal{C}_0)|} \geq \eta \\
&= 0; \quad if \ \frac{|\widehat{H}_{\mathrm{s}\to\mathrm{meta}\to\mathrm{mr}}(\mathbf{r}_\mathrm{m}, \omega; \mathcal{C}_1)|}{|\widehat{H}_{\mathrm{s}\to\mathrm{meta}\to\mathrm{mr}}(\mathbf{r}_\mathrm{m}, \omega; \mathcal{C}_0)|} < \eta
\end{aligned}$$

Note that the information bit "1" corresponds to the case that the $\mathcal{C}_1$ is chosen such that the stray wireless signal carrying the coding information of the metasurface has its energy focused around $\mathbf{r}_\mathrm{m}$. In contrast, the information bit "0" means that the $\mathcal{C}_1$ is chosen such that the wireless signal acquired by the receiver at $\mathbf{r}_\mathrm{m}$ has relatively lower energy level.

- <u>Binary Phase Shift Keying (BPSK)</u>

$$I_{\mathrm{out}} = 1; \quad \text{if } phase\left(\widehat{H}_{\mathrm{s}\to\mathrm{meta}\to\mathrm{mr}}(\mathbf{r}_{\mathrm{m}}, \omega; \mathcal{C}_1)\right) = 0$$

$$= 0; \quad \text{if } phase\left(\widehat{H}_{\mathrm{s}\to\mathrm{meta}\to\mathrm{mr}}(\mathbf{r}_{\mathrm{m}}, \omega; \mathcal{C}_0)\right) = \pi$$

In addition, for the cases of "0" and "1", the energies of the stray wireless signals reflected from the metasurface need to be well focused around the receiver at $\mathbf{r}_{\mathrm{m}}$, i.e.,

$$|\widehat{H}_{\mathrm{s}\to\mathrm{meta}\to\mathrm{mr}}(\mathbf{r}_{\mathrm{m}}, \omega; \mathcal{C}_1)| \approx |\widehat{H}_{\mathrm{s}\to\mathrm{meta}\to\mathrm{mr}}(\mathbf{r}_{\mathrm{m}}, \omega; \mathcal{C}_0)|.$$

- <u>Quadrature Phase Shift Keying (QPSK)</u>

$$I_{\mathrm{out}} = 11; \quad \text{if } phase\left(\widehat{H}_{\mathrm{s}\to\mathrm{meta}\to\mathrm{mr}}(\mathbf{r}_{\mathrm{m}}, \omega; \mathcal{C}_3)\right) = 0$$

$$= 10; \quad \text{if } phase\left(\widehat{H}_{\mathrm{s}\to\mathrm{meta}\to\mathrm{mr}}(\mathbf{r}_{\mathrm{m}}, \omega; \mathcal{C}_2)\right) = \pi/2$$

$$= 01; \quad \text{if } phase\left(\widehat{H}_{\mathrm{s}\to\mathrm{meta}\to\mathrm{mr}}(\mathbf{r}_{\mathrm{m}}, \omega; \mathcal{C}_1)\right) \geq \pi$$

$$= 00; \quad \text{if } phase\left(\widehat{H}_{\mathrm{s}\to\mathrm{meta}\to\mathrm{mr}}(\mathbf{r}_{\mathrm{m}}, \omega; \mathcal{C}_0)\right) = 3\pi/2$$

As opposed to the case of binary modulation scheme, the QPSK involves the 4-phase quantization. For this case, four different coding patterns of the metasurface need to be optimized such that the phases of $\widehat{H}_{\mathrm{s}\to\mathrm{meta}\to\mathrm{mr}}(\mathbf{r}_{\mathrm{m}}, \omega; \mathcal{C}_{\mathrm{i}})$ ($i =$ 0, 1, 2, and 3) can be well distinguished. In addition, energy constraints on $\widehat{H}_{\mathrm{s}\to\mathrm{meta}\to\mathrm{mr}}(\mathbf{r}_{\mathrm{m}}, \omega; \mathcal{C}_{\mathrm{i}})$ are needed, i.e.,

$$|\widehat{H}_{\mathrm{s}\to\mathrm{meta}\to\mathrm{mr}}(\mathbf{r}_{\mathrm{m}}, \omega; \mathcal{C}_3)| \approx |\widehat{H}_{\mathrm{s}\to\mathrm{meta}\to\mathrm{mr}}(\mathbf{r}_{\mathrm{m}}, \omega; \mathcal{C}_2)|.$$
$$\approx |\widehat{H}_{\mathrm{s}\to\mathrm{meta}\to\mathrm{mr}}(\mathbf{r}_{\mathrm{m}}, \omega; \mathcal{C}_1)|$$
$$\approx |\widehat{H}_{\mathrm{s}\to\mathrm{meta}\to\mathrm{mr}}(\mathbf{r}_{\mathrm{m}}, \omega; \mathcal{C}_0)|$$

Supplementary Note 3. Information encoding in MBWC

Here, we would like to provide some helpful insights into the information encoding for the proposed MBWC. To this end, it is crucial to perform the analysis on Supplementary Equation 5. More specifically, we need to provide an in-depth investigation of $\sum_n \sigma_n(\mathcal{C}) G(\mathbf{r}_{\mathrm{m}}, \mathbf{r}_n) G(\mathbf{r}_n, \mathbf{r}_1)$. For simplicity, we assume that the background medium is vacuum, and that both the unknown wireless signal source and the master receiver are in the far-field region of the metasurface. As a result, we have

$$G(\mathbf{r}_{\mathrm{m}}, \mathbf{r}_n) \approx \frac{\exp(jk_0 r_m)}{4\pi r_m} \exp\left(-j\hat{\mathbf{k}}_{\mathbf{m}} \cdot \mathbf{r}_n\right)$$

and $G(\mathbf{r}_1, \mathbf{r}_n) \approx \frac{\exp(jk_0 r_1)}{4\pi r_1} \exp(-j\hat{\mathbf{k}}_1 \cdot \mathbf{r}_n)$

Then, we have

$$\sum_n \sigma_n(\mathcal{C}) G(\mathbf{r}_\mathrm{m}, \mathbf{r}_n) G(\mathbf{r}_n, \mathbf{r}_1) \approx \frac{\exp(jk_0(r_\mathrm{m}+r_1))}{16\pi^2 r_1 r_\mathrm{m}} \sum_n \sigma_n(\mathcal{C}) \exp(-j(\hat{\mathbf{k}}_\mathrm{m} + \hat{\mathbf{k}}_1) \cdot \mathbf{r}_n)$$
$$\approx \frac{\exp(jk_0(r_\mathrm{m}+r_1))}{16\pi^2 r_1 r_\mathrm{m}} \sum_n \sigma_n(\mathcal{C}) \exp(-j(\hat{\mathbf{k}}_{\rho,\mathrm{m}} + \hat{\mathbf{k}}_{\rho,1}) \cdot \boldsymbol{\rho}_n) \quad (14)$$

Herein, $\mathbf{r}_n = (\boldsymbol{\rho}_n, 0)$, $\hat{\mathbf{k}}_1 = (\hat{\mathbf{k}}_{\rho,1}, \hat{\mathbf{k}}_{z,1})$, and $\hat{\mathbf{k}}_\mathrm{m} = (\hat{\mathbf{k}}_{\rho,\mathrm{m}}, \hat{\mathbf{k}}_{z,1})$.

Furthermore, we take the spectral representation of $\sigma(\mathcal{C})$ as following, i.e.,

$$\sigma_n(\mathcal{C}) = \sum_{\boldsymbol{\varkappa}} \tilde{\sigma}(\boldsymbol{\varkappa}) \exp(j\boldsymbol{\varkappa} \cdot \boldsymbol{\rho}_n) \quad (15)$$

Substituting Supplementary Equation 15 into Supplementary Equation 14 leads to

$$\sum_n \sigma_n(\mathcal{C}) G(\mathbf{r}_\mathrm{m}, \mathbf{r}_n) G(\mathbf{r}_n, \mathbf{r}_1)$$
$$\approx \frac{\exp(jk_0(r_m+r_1))}{16\pi^2 r_1 r_m} \sum_{\boldsymbol{\varkappa}} \tilde{\sigma}(\boldsymbol{\varkappa}) \left[\sum_n \exp(j(\boldsymbol{\varkappa} - \hat{\mathbf{k}}_{\rho,\mathrm{m}} - \hat{\mathbf{k}}_{\rho,1}) \cdot \boldsymbol{\rho}_n)\right]$$
$$\approx \frac{\exp(jk_0(r_m+r_1))}{16\pi^2 r_1 r_m} \sum_{\boldsymbol{\varkappa}} \tilde{\sigma}(\boldsymbol{\varkappa}) \, \mathrm{sinc}(\boldsymbol{\varkappa} - \hat{\mathbf{k}}_{\rho,1} - \hat{\mathbf{k}}_{\rho,\mathrm{m}}) \quad (16)$$

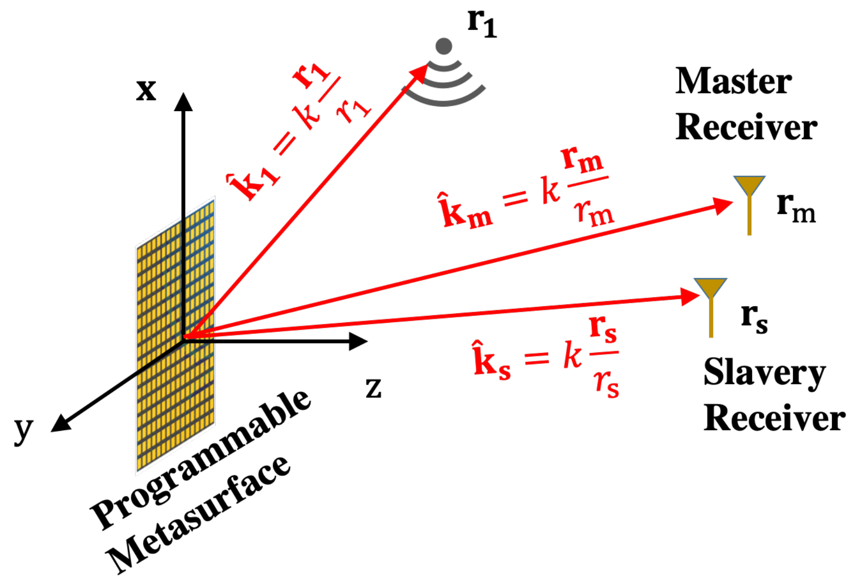

Supplementary Figure 2. Sketched maps for deriving Supplementary Equation 14.

Herein, *sinc* is the standard sinc function, and its width is determined by the size of metasurface aperture. Supplementary Equation 16 renders us **an important conclusion that, wherever the non-cooperative wireless source is, its energy can be well focused**

toward the master receiver at $\mathbf{r}_\mathrm{m}$ by choosing a suitable control coding pattern of the metasurface, i.e., $\tilde{\sigma}(\boldsymbol{\varkappa})$. Then, it is safe to say that the demodulated signal $\sum_n \sigma_n(\mathcal{C})G(\mathbf{r}_\mathrm{m},\mathbf{r}_n)G(\mathbf{r}_n,\mathbf{r}_1)$ is approximately independent of the location of the unknown wireless source by controlling the coding pattern of the metasurface. For this reason, the factor $G(\mathbf{r}_n,\mathbf{r}_1)$ has been suppressed in the expression of the demodulated signal $\widehat{H}_{\mathrm{s}\to\mathrm{meta}\to\mathrm{mr}}$, i.e., $\widehat{H}_{\mathrm{s}\to\mathrm{meta}\to\mathrm{mr}} \approx A\sum_n \sigma_n(\mathcal{C})G(\mathbf{r}_\mathrm{m},\mathbf{r}_n)$, where A is a complex-valued constant. Of course, it is not difficult to estimate the orientation of the unknown wireless source by using a standard beam scanning technique.

From the above discussions, we can see that the coherence function of the random-like wireless signals acquired by two or more antennas is determined by the coding pattern of the programmable metasurface. Explicitly generalizing the above discussion to a complex propagation environment is very challenging since typically no tractable models are available for such scenarios, i.e. no analytical or numerical expression for the Green's function is available. Nonetheless, it is possible to leverage machine-learning tools to train an artificial neural network to approximate the Green's function, as in Ref.[2]. Once such a learned forward model has been obtained, the above solution can be directly applied for the purpose of MBWC.

Supplementary Note 4. Optimization of the information-carrying control coding pattern

We here discuss the inverse-design of the information-carrying control coding patterns of the programmable metasurface. For simplicity, we consider the binary modulation as an illustrative example; however, the reported methods and results can be readily generalized for multi-bit modulation in a straightforward manner.

As discussed in **Supplementary Note 1**, two "distinguishable" control coding patterns of the metasurface (referred to as pattern-1 $\mathcal{C}_0$ and pattern-2 $\mathcal{C}_1$) are optimized such that their associated radiation beams are visible to one receiver (called the master receiver), and meanwhile are invisible to another receiver (called the slavery receiver). Here, we mean by the "distinguishability" that the acquired wireless signals reflected from the metasurface with $\mathcal{C}_0$ can be readily distinguished from those with $\mathcal{C}_1$ by a classifier. Moreover, we mean by "visibility" that the intensity of the wireless signal reflected from the metasurface

is focused around the master receiver. In terms of the methods outlined in **Supplementary Note 1**, we can see that the stray wireless signal carrying the digital information encoded into the metasurface can be readily demodulated, and thus the digital information can be easily decoded.

Formally, we can formulate the inverse-design of the distinguishable control coding pattern of the metasurface for the proposed MBWC into a constrained optimization problem, in particular,

$$\min_{\mathcal{C}_0,\mathcal{C}_1}\left\{\left\||\widehat{H}_{\text{s}\to\text{meta}\to\text{mr}}(:,\mathcal{C}_0)|-|\boldsymbol{E}^{\text{des}}|\right\|_2^2+\left\||\widehat{H}_{\text{s}\to\text{meta}\to\text{mr}}(:,\mathcal{C}_1)|-|\boldsymbol{E}^{\text{des}}|\right\|_2^2\right\} \tag{17}$$

s.t., $\{\widehat{H}_{\text{s}\to\text{meta}\to\text{mr}}(:,\mathcal{C}_0)\}$ and $\{\widehat{H}_{\text{s}\to\text{meta}\to\text{mr}}(:,\mathcal{C}_1)\}$ are as much distinguishable from each other as possible.

Herein, $\widehat{H}_{\text{s}\to\text{meta}\to\text{mr}}$ is a $2\times F$-length complex-valued column vector. $\boldsymbol{E}^{\text{des}}$, a $2\times F$-length complex-valued column vector, represents the desired wireless signals, in particular, the signal component of $\boldsymbol{E}^{\text{des}}$ acquired by the master receiver are visible to the metasurface, while the components corresponding to the slavery receiver are invisible to the metasurface. Moreover, F indicates the number of discrete frequency points of the acquired wireless signals, if the non-cooperative wireless signal considered has certain operational frequency bandwidth.

Here, we heuristically introduce two simple algorithms for solving Supplementary Equation 17, which can be derived from Supplementary Equation 16.

Algorithm 1. Inverse-design of the coding pattern for the MBWC ASK modulation.

Ideally, the required phase of the metasurface meta-atom at $\mathbf{r}_{ij}=(x_i,y_j,0)$is:

$$\varphi_{ideal\_ASK}(x_i,y_j)=\arg\{\textstyle\sum_{n=1}^{N}[U_0^n exp(-jk_0|\mathbf{r}_n-\mathbf{r}_{ij}|)]\} \tag{18}$$

where N is the total number of specified positions at which the energy of stray Wi-Fi signals reflected from the metasurface is focused. Accordingly, at these positions, the amplitudes of ambient stray Wi-Fi signals are modulated by the digital information encoded into the metasurface, and $\mathbf{r}_n$ represents the coordinate of the nth position of interest. For the binary ASK modulation, the relative intensity of modulated Wi-Fi signals at each desirable

position can be controlled by $U_0^n = 0$ or 1. Since the meta-atom has two states, i.e., "ON" and "OFF" state, the ideal phase characterized by Supplementary Equation 18 needs to be discretized accordingly, i.e., according to the following equation:

$$\varphi_d(x_i, y_j) = \begin{cases} 0°, & -90° < \varphi_{ideal\_ASK}(x_i, y_j) \le 90° \\ 180°, & \text{otherwise} \end{cases} \quad (19)$$

Algorithm 2. Inverse-design of the coding pattern for the MBWC PSK modulation.

For the case of M-phase PSK, Supplementary Equation 18 can be heuristically extended into the following expression, i.e.,

$$\varphi_{idea\_PSK}(x_i, y_j; p) = \arg\{\sum_{n=1}^{N}[exp(-jk_0|\mathbf{r}_n - \mathbf{r}_{ij}|)e^{j\Delta\varphi_{pn}}]\} \quad (20)$$
$$p = 1,2,\dots,M$$

Here, $\Delta\varphi_{pn}$ denotes the desirable phase at the focusing location of $\boldsymbol{r}_n$. Specifically, for the M-phase PSK, $\Delta\varphi_{pn}$ has M choices, i.e., $\Delta\varphi_{pn} = 2\pi(p-1)/M$, $(p = 1,2,\dots,M)$. Again, considering that the meta-atom has two digital states of ON and OFF, Supplementary Equation 20 needs to be quantized as done in Supplementary Equation 19. In particular,

$$\varphi_d(x_i, y_j; p) = \begin{cases} 0°, & -90° < \varphi_{idea\_PSK}(x_i, y_j; p) \le 90° \\ 180°, & \text{otherwise} \end{cases} \quad (21)$$

Supplementary Note 5. Design of the programmable coding metasurface.

We elaborate on electronically-controllable binary-phase meta-atom in terms of design parameters, fabrication and tests. As shown in **Supplementary Figure 3a**, the meta-atom is composed of two substrate layers: the top substrate is F4B with the relative permittivity of 2.55 and loss tangent of 0.0015, and the bottom substrate is FR4. The top square patch, which is responsible for reflecting incoming electromagnetic (EM) waves, is integrated with a SMP1345-079LF PIN diode connected to the ground plane via a hole. We choose the SMP1345-079LF diode because it has relatively low insertion loss (< 0.2dB) and high isolation>13dB) in the desired frequency band. A TDK chip inductor with inductance L = 33nH (MLK1005S33NJT000) is used to suppress the AC coupling to ground. We examine the EM performance of the meta-atom numerically and experimentally. In numerical

simulations, we use a commercial full-wave EM simulator, CST Microwave Transient Simulation Package 2017. Additionally, a series lumped-parameter circuit is deliberately chosen to model the PIN diode. When the diode is switched ON, it is represented by a 0.7nH inductor in series with a 2Ω resistor. By contrast, when the diode is switched OFF, it is modeled by a 1.8pF capacitor in series with a 0.7nH inductor. The meta-atom has been designed, fabricated and tested. The fabricated sample and waveguide-based measurement setup are given in **Supplementary Figure 3b**, where a standard waveguide to coaxial adapter A-INFO 430WCAS is used. The simulation and experiment results of meta-atom are compared in **Supplementary Figure 3c-d**, when the meta-atoms are mounted inside the waveguide. We observe that the reflection phase of the meta-atom experiences180°±20° phase difference when the PIN diode is switched from ON (OFF) to OFF (ON) in the frequency range 2.44-2.52 GHz. The phase change can be accomplished by switching the external DC voltage applied to the PIN diode from 3.3V to 0V. It is worth mentioning that the measured results using waveguide method cannot be compared directly with the simulation results under unit cell boundaries due to the non-strict periodic environment of waveguide. However, the measured results can still be used to verify the performance of meta-atom.

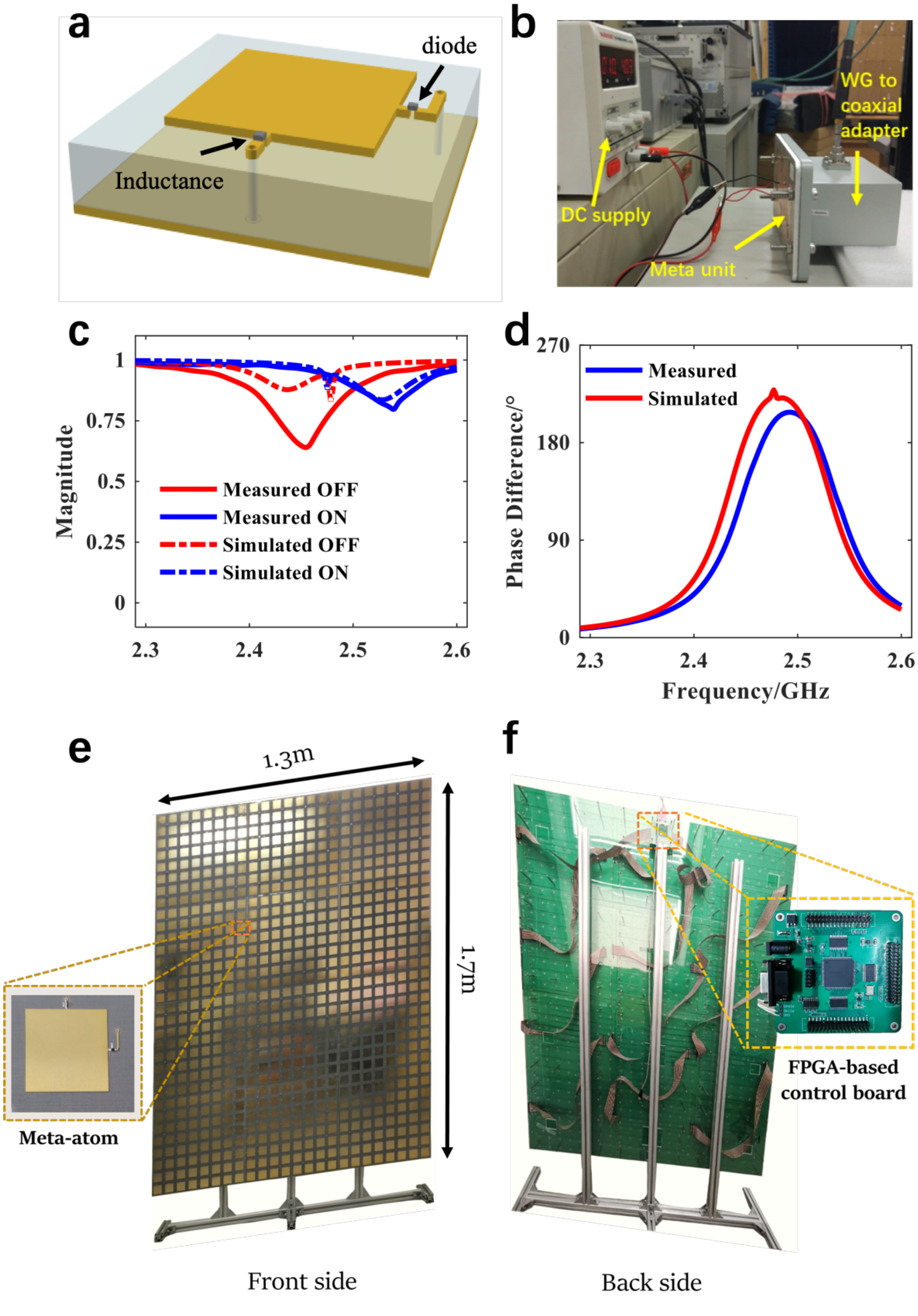

Supplementary Figure 3. **a**, Sketched map of designed meta-atom. **b,** Experimental setup of waveguide-based test on the designed meta-atom. **c-d**, Experimental and simulated results of magnitude-frequency and phase-frequency responses of designed meta-atom, respectively. **e-f,** Picture of designed programmable coding metasurface with size of 1.3m x 1.7m, where the front side and back side pictures are provided. In this figure, both the designed meta-atom and FPGA-based control board have been inserted in **e** and **f**, respectively.

The designed programmable metasurface works around 2.412 GHz, consistent with the commodity Wi-Fi frequency. The metasurface is composed of independently-controllable 32×24 meta-atoms. Since each meta-atom has a size of $54\times54\text{mm}^2$, the whole metasurface has size of $1.7\times1.3\text{m}^2$ in total. We remark that the whole metasurface is composed of 3×4 identical panels due to the restriction of fabrication, and each panel has 8 x 8 meta-atoms. The whole programmable metasurface is electronically controlled with a FPGA-based Micro-Control-Unit (MCU). A FPGA chip is used to distribute all commands to 768 PIN diodes. To achieve the real-time and flexible controls of 768 PIN diodes soldered in the programmable metasurface, a MCU with size of $90\times90\text{mm}^2$ is designed and assembled on the upper rear of the metasurface. The MCU is responsible for dispatching all commands sent from a master computer subject to one common clock (CLK) signal. In our work, the adopted CLK is 50MHz, and the switching time of PIN diode is about 10μs each cycle. Then MCU will send the commands over 24 independent branch channels, leading to almost real-time manipulations of all PIN diodes. In addition, 768 red-color LEDs are soldiered to indicate the status of the associated PIN diodes, in particular, to indicate clearly whether the PIN diode works well or not.

Supplementary Note 6. Performance of the proposed MBWC

We evaluate the performance of the MBWC prototype by taking the three-channel BASK MBWC as illustrative examples.

1. Intensity ratio R

Here, we define the intensity ratio R as $|\widehat{H}_{\text{s}\to\text{meta}\to\text{mr}}|$, i.e., is $R=|\widehat{H}_{\text{s}\to\text{meta}\to\text{mr}}|=\left|\frac{H_{\text{s}\to\text{meta}\to\text{mr}}}{H_{\text{s}\to\text{sr}}}\right|$. Here, a set of experimental results are provided to verify the claim that the energy of stray Wi-Fi waves can be well allocated to the desired spot by controlling the coding pattern of the metasurface. In our experiments, a commercial Wi-Fi router is placed at (0m, 0m,1.21m), a slavery receiver is located at (-0.16m, 0.12m,1m), and three master receivers are respectively located at R (0m, 0.5m, 2.28m), G (0m, 0m, 2.28m) and B (0.7m, 0.6m, 2.28m). **Supplementary Figure 4** depict the comparison of intensity ratio R between the proposed signal model and the experimental measurements at aforementioned three locations when the programmable metasurface is configured with different control coding

patterns. It is obvious that the trend of intensity ratio *R* predicted by the analytical model match well with the measured results. More importantly, it can be immediately observed that the intensity ratio R can be remarkably bigger than 1 by controlling the coding pattern of the metasurface, implying that the energy of stray Wi-Fi waves carrying the Alice's information can be well allocated towards the spot of interest.

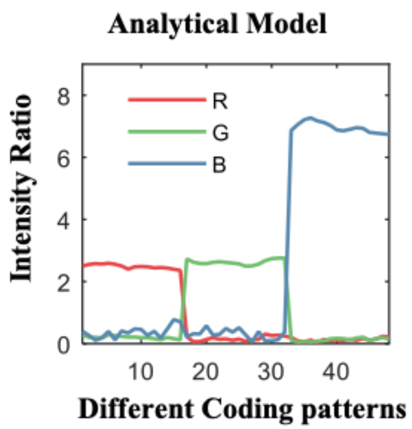

Supplementary Figure 4. Comparison of the intensity ratio R between the proposed analytical model (left) and the measurements (right), where 50 different coding patterns of the metasurface are considered.

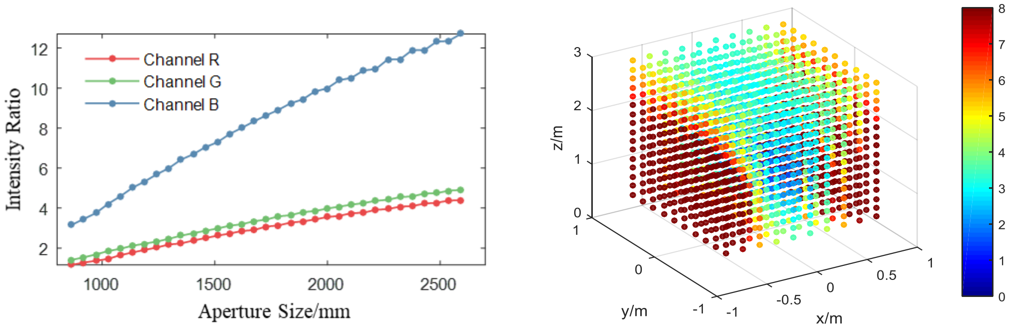

Supplementary Figure 5. (Left) numerical results about the relationship between the intensity ratio R and the aperture size of metasurface, (right) 3-D distribution of intensity ratio.

Here, we give two marks about the intensity ratio. First, the relationship between the intensity ratio and aperture size of metasurface is investigated. Corresponding numerical results are provided in **Supplementary Figure 5a**. It can be expected that, when the metasurface is configured with suitable coding patterns, the bigger the aperture size of metasurface is, the bigger the intensity ratio is. This does make sense because more energy

of the Wi-Fi signals can be well harvested if the metasurface with bigger aperture size is used. Second, we consider the influence of focus locations of metasurface on the intensity ratio. As shown in **Supplementary Figure 5b**, the focus spots are represented by filled circles and the color of circles denotes the predicted intensity ratio R. It is worth mentioning that we limit the color axis less than 8 to facilitate the display of figure. As expected, the intensity ratio R becomes large when the position of master receiver is away from yoz plane where the direct wave from Wi-Fi source is fairly strong since the Wi-Fi source is polarized along the x direction.

2. Channel strength

As outlined previously, one of crucial issues to the MBWC is that the master receiver is "visible" to the metasurface, i.e., this receiving antenna sits within the local focusing spot of the metasurface. It is clear that the MBWC relies on a critical property of the programmable metasurface that the energy of the stray wireless signals can be reallocated and enhanced towards the desirable local spot without extra energy consumption. To examine this property, we introduce the concept of the channel strength for the proposed MBWC, which is defined as the intensity average of the wave within the considered local focusing spot. Apparently, the SNR of the modulated and demodulated signal is proportional to the channel strength. In particular, the stronger the channel strength is, the higher the SNR is.

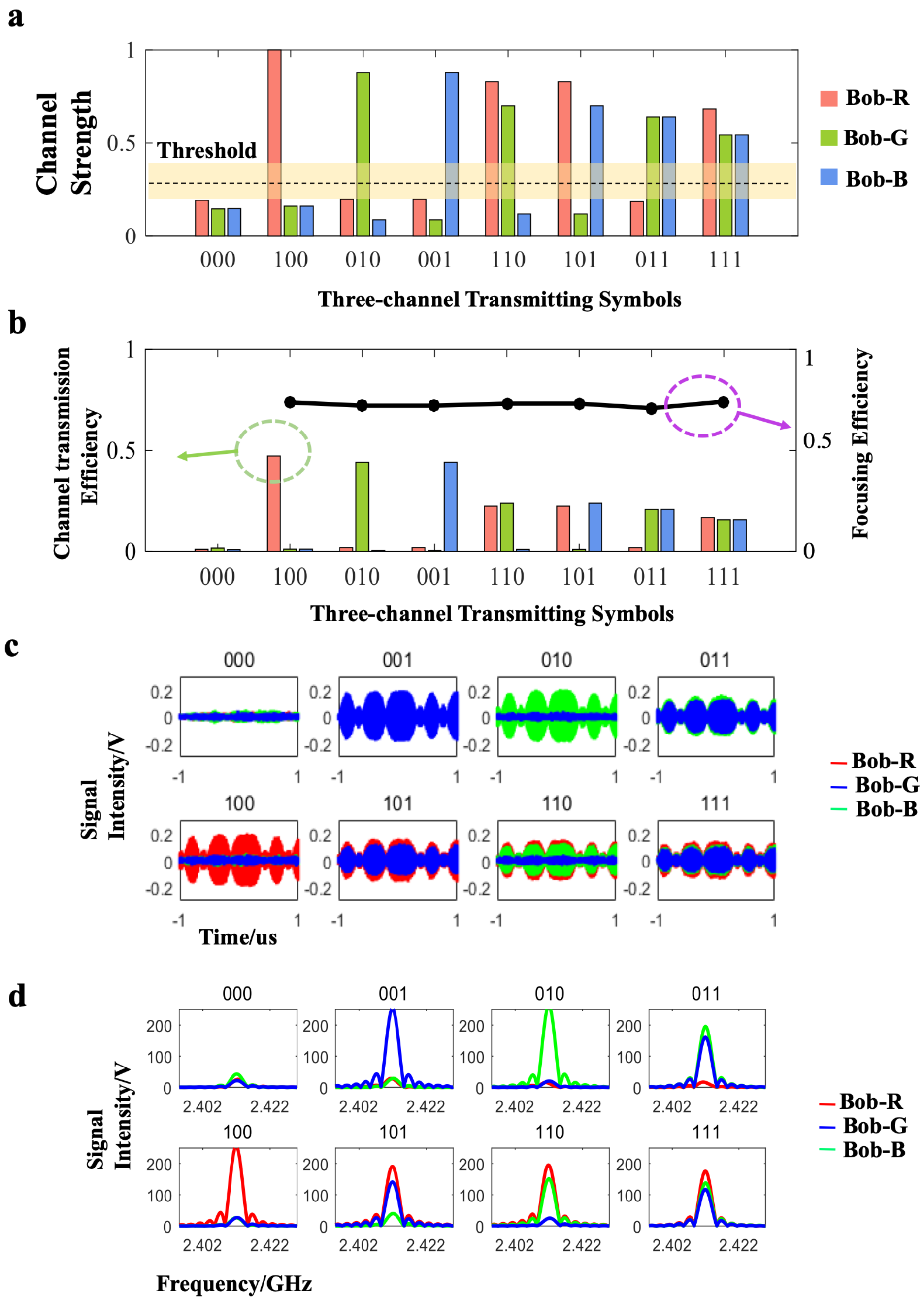

Supplementary Figure 6. a, The channel strengths for different three-channel transmitting symbols. **b**, The channel transmission efficiencies and the focusing efficiencies of the designed programmable metasurface. **c,** The 2μs-long commodity Wi-Fi signals for different three-channel transmitting symbols. **d,** The amplitude-frequency representations of **c**. The experimental setup of these results is the same as that used in Figure 3 in main text.

A set of experiments have been conducted to demonstrate the channel strengths of the proposed MBWC. The channel strengths of three channels, corresponding to the optimized

8 coding patterns shown in **Figure 3**, have been plotted in different colors in **Supplementary Figure 6a**. In these figures, the channel strengths are normalized by the maximum channel strength of the transmitting symbol '100'. From these figures, it is clear that the channel strength is inverse proportional to the number of the efficient communication channels, which makes sense since the total energy of the stray Wi-Fi signals reflected from the metasurface are fixed, and thus the increase of communication channels will directly give rise to the reduce of channel strengths. Therefore, as long as a proper decision threshold is used, the digital symbols transmitted from Alice can be easily identified from the intensities of ambient stray Wi-Fi signals. Here, we would like to point out that the channel strength can be improved by increasing the metasurface aperture, as shown in **Supplementary Figure 5**.

3. Channel transmission efficiency

The channel transmission efficiency is defined as the ratio of the total energy within the focusing spot to that in the whole receiving plane. Experimental results have been reported in **Supplementary Figure 6**. Meanwhile, the corresponding focusing efficiencies of the metasurface have been provided as well. The focusing efficiency of the metasurface is defined as the ratio of the total energy within all focusing spots to that in the receiving plane, which almost keeps constant in all combinations of digital symbols due to the energy conversation. From these experiments, we can clearly see that the highest and lowest channel transmission efficiency are 47.25% and 15.68%, respectively, and that the focusing efficiency of the metasurface is not influenced by the channel number.

4. Effect on the communication quality from the communication distance

Here, we would like to examine the effect on the quality of the MBWC from the distance of the Wi-Fi router away from the metasurface. To that end, a set of experiments have been conducted, where a full-color image of 100 pixels has been transferred from Alice to Bob-R, Bob-G and Bob-B using the proposed MBWC prototype. **Supplementary Figure 7a** shows the measured amplitudes the passively modulated Wi-Fi signals by Bob-R, Bob-G and Bob-B, where the Wi-Fi router is placed at different distances of z=1.21m, z=3m, z=4m and z=5m. In these figures, the experimentally measured amplitude has been normalized

to the maximum value of all channels. The recovered images corresponding to different Wi-Fi router's locations are shown in **Supplementary Figure 7b**, in which the amplitude decision threshold is set to be 0.28. As is shown in **Supplementary Figure 7b**, the $|\widehat{H}_{\mathrm{s\to meta\to mr}}(:,\mathcal{C}_0)|$ and $|\widehat{H}_{\mathrm{s\to meta\to mr}}(:,\mathcal{C}_1)|$ becomes more and more entangled when the distance of Wi-Fi router away from the programmable metasurface increase. Since the component of stray Wi-Fi signal carrying the Alice's information become weaker with the growth of the Wi-Fi router's location, i.e., $|\widehat{H}_{\mathrm{s\to meta\to mr}}(:,\mathcal{C}_0)|$ become weaker, thus, $\widehat{H}_{\mathrm{s\to meta\to mr}}(:,\mathcal{C}_0)$ cannot be well demodulated due to the noise corruption. In addition, **Supplementary Table I** reports the bit error rates in these channels as the Wi-Fi router's location changes. Channel R has the lowest bit error rate because it has higher SNR than G and B since it is closer to the Wi-Fi source. Although the positions of receiving antenna G and B are symmetrical, its BERs are clearly different, which may due to the complex environment.

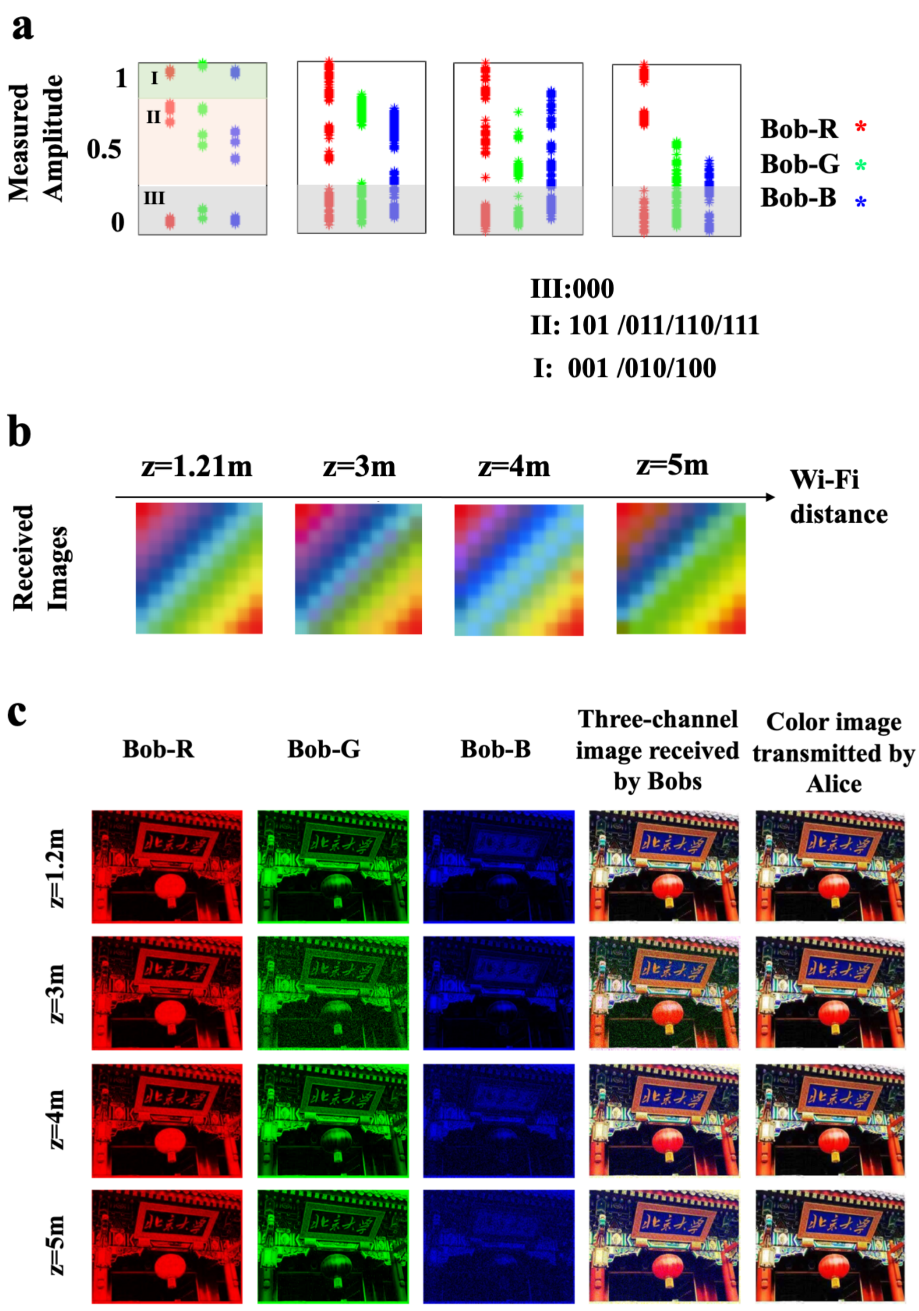

Supplementary Figure 7. The influence of Wi-Fi router's distance on the BER (bit error rate) of the proposed three-channel ASK MBWC. **a**, The experimental amplitude of three-channel passively modulated Wi-Fi signals when Wi-Fi router sits at different distances of z=1.21m, z=3m, z=4m and z=5m. All the measured amplitude has been normalized to the measured maximum of every channel. **b**, The recovered color-scale images corresponding to **a**, where the image for z=1.21m can be treated as the perfect image transmitted by Alice, since it has almost zero BER. **c,** the images recovered separately by Bob-R, Bob-G, and Bob-B, and the recovered color-scale image by Bobs, when the Wi-Fi router is at different distances. This set of results corresponds to those used in Figure 3 in main text.

Supplementary Table I The BER of three-channel ASK MBWC

[redacted]	[redacted]	[redacted]	[redacted]
z=1.2m	0.00%	0.00%	0.00%
z=3.0m	0.00%	10.09%	0.00%
z=4.0m	0.00%	0.13%	13.38%
z=5.0m	0.00%	0.25%	22.13%

a, signal model of MBWC encoding &modulation

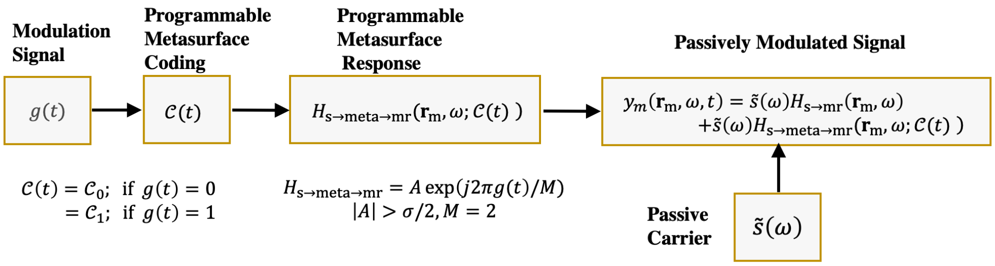

b, signal model of MBWC decoding & demodulation

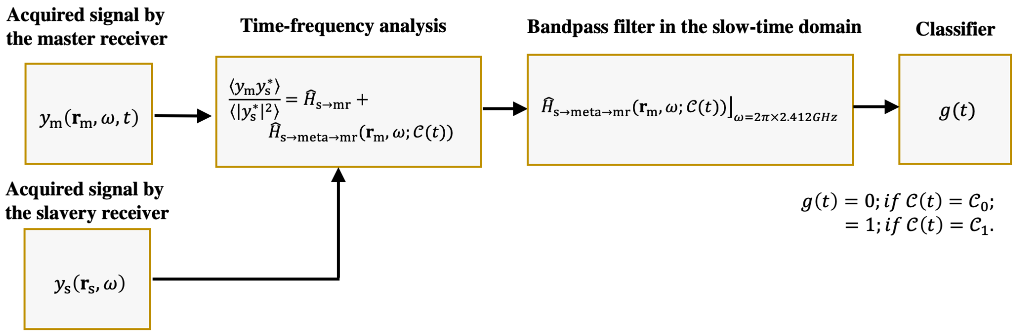

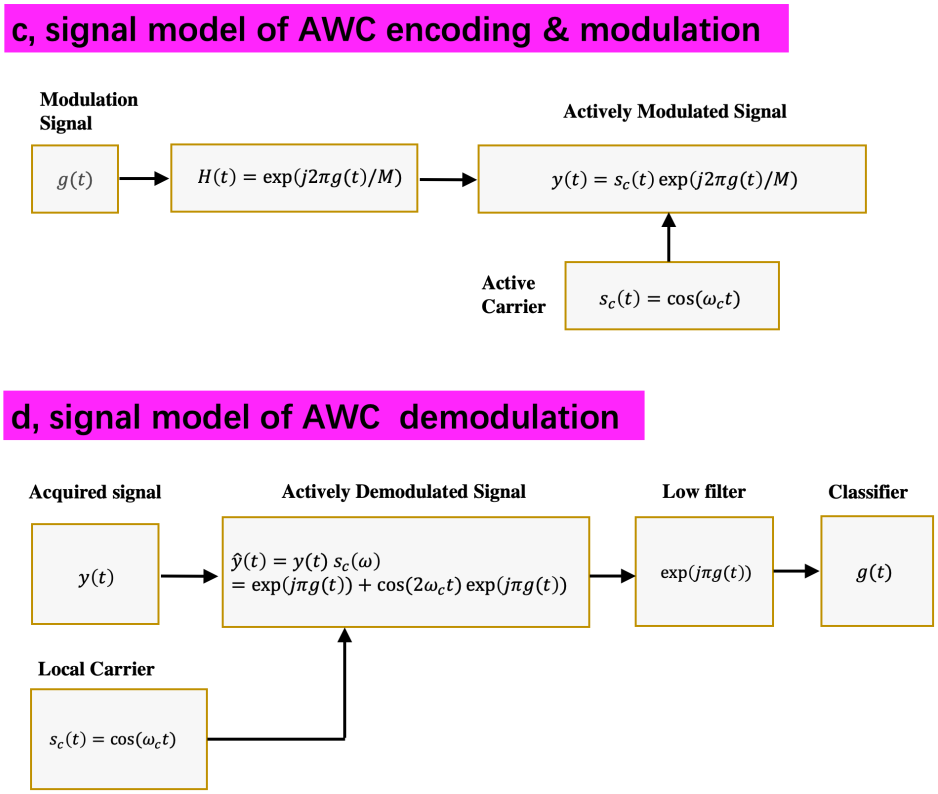

Supplementary Figure 8. The signal models of the modulation and demodulation of the proposed MBWC. Here, the binary phase modulation and demodulation are considered for the illustration purpose. For comparison, the corresponding modulation and demodulation models for the AWC are also provided in c and d.

Supplementary Note 7. Procedure of MBWC demodulation and decoding

The procedure of information retrieval of the proposed BPSK MBWC is summarized in **Supplementary Figure 8**. The 2us-length Wi-Fi signals acquired by the master and slavery receivers are plotted in **Supplementary Figure 9a**. To retrieve the information symbols encoded into the metasurface at Alice side, several critical operations are implemented.

Step 1. Time-frequency analysis.

A time-frequency analysis is made by calculating $\frac{\langle y_{\mathrm{m}}(\omega,\mathcal{C}(t))\, y_{\mathrm{s}}^{*}(\omega,\mathcal{C}(t))\rangle}{\langle |y_{\mathrm{s}}^{*}(\omega,\mathcal{C}(t))|^2\rangle}$, where the time t denotes the slow time, and the angular frequency ω arises from the fast time. Herein, y_{m} and y_{s} denote the ambient stray Wi-Fi signals acquired by the master and slavery antenna,

respectively. With such analysis, the unwanted effect on the MBWC demodulation from the unknown Wi-Fi signal can be easily calibrated out. Corresponding experimental results of $\frac{\langle y_{\mathrm{m}} y_{\mathrm{s}}^* \rangle}{\langle |y_{\mathrm{s}}^*|^2 \rangle}$ for different transmitting symbols are plotted in **Supplementary Figure 9b**. Note that the windowed faster Fourier transfer (wFFT) is needed for processing the acquired raw time-domain Wi-Fi signals before running $\frac{\langle y_{\mathrm{m}} y_{\mathrm{s}}^* \rangle}{\langle |y_{\mathrm{s}}^*|^2 \rangle}$, and that the width of the wFFT is the duration of a MBWC digital symbol.

Note that $\frac{\langle y_{\mathrm{m}}(\omega,\mathcal{C}(t)) y_{\mathrm{s}}^*(\omega,\mathcal{C}(t)) \rangle}{\langle |y_{\mathrm{s}}^*(\omega,\mathcal{C}(t))|^2 \rangle}$ is a function of Wi-Fi's frequency. For simplicity, we would like to pick up the value of $\left.\frac{\langle y_{\mathrm{m}}(\omega,\mathcal{C}(t)) y_{\mathrm{s}}^*(\omega,\mathcal{C}(t)) \rangle}{\langle |y_{\mathrm{s}}^*(\omega,\mathcal{C}(t))|^2 \rangle}\right|_{\omega=2\pi\times 2.412\ \mathrm{GHz}}$ for any slow time, since it corresponds to the maximum of $\frac{\langle y_{\mathrm{m}}(\omega,\mathcal{C}(t)) y_{\mathrm{s}}^*(\omega,\mathcal{C}(t)) \rangle}{\langle |y_{\mathrm{s}}^*(\omega,\mathcal{C}(t))|^2 \rangle}$ over the whole frequency range. Then, for the passive transmission of a color-scale image of 100 pixels shown in **Supplementary Figure 9c,** the constellation of $\left.\frac{\langle y_{\mathrm{m}}(\omega,\mathcal{C}(t)) y_{\mathrm{s}}^*(\omega,\mathcal{C}(t)) \rangle}{\langle |y_{\mathrm{s}}^*(\omega,\mathcal{C}(t))|^2 \rangle}\right|_{\omega=2\pi\times 2.412\ \mathrm{GHz}}$ is plotted in **Supplementary Figure 9c**.

Step 2. Direct-arrival removal.

As shown in Supplementary Equation 5, the coherence function $\left.\frac{\langle y_{\mathrm{m}}(\omega,\mathcal{C}(t)) y_{\mathrm{s}}^*(\omega,\mathcal{C}(t)) \rangle}{\langle |y_{\mathrm{s}}^*(\omega,\mathcal{C}(t))|^2 \rangle}\right|_{\omega=2\pi\times 2.412\ \mathrm{GHz}}$ has two terms, in which the first term represents the direct-arrival response, and it is independent of the slow time. In order to retrieve the digital information encoded into the metasurface, the first term needs to be removed.

We perform the media filter of $\left.\frac{\langle y_{\mathrm{m}}(\omega,\mathcal{C}(t)) y_{\mathrm{s}}^*(\omega,\mathcal{C}(t)) \rangle}{\langle |y_{\mathrm{s}}^*(\omega,\mathcal{C}(t))|^2 \rangle}\right|_{\omega=2\pi\times 2.412\ \mathrm{GHz}}$ with respect to the slow time *t* in order to arrive at the demodulated signal $\widehat{H}_{\mathrm{s}\to\mathrm{meta}\to\mathrm{mr}}(:,\mathcal{C}(t))$, by which the direct arrival $\widehat{H}_{\mathrm{s}\to\mathrm{mr}}$ can be easily removed. Corresponding experimental results have been shown in **Supplementary Figure 9d**.

Step 3. Phase-difference-error mitigation.

From Supplementary Equation 5, the demodulated signal is

$$\widehat{H}_{\mathrm{s}\to\mathrm{meta}\to\mathrm{mr}} \approx \left(G(\mathbf{r}_{\mathrm{s}}, \mathbf{r}_1)\right)^* \sum_n \sigma_n(\mathcal{C}) G(\mathbf{r}_{\mathrm{m}}, \mathbf{r}_n) G(\mathbf{r}_n, \mathbf{r}_1).$$

$$\approx \frac{\exp(jk_0(r_\mathrm{m}-r_\mathrm{s}+r_1))}{16\pi^2 r_1 r_\mathrm{m} r_\mathrm{s}} \exp\left(j\hat{\mathbf{k}}_\mathrm{s}\cdot\mathbf{k}_1\right)\sum_n \sigma_n(\mathcal{C})\exp\left(-j(\hat{\mathbf{k}}_{\rho,\mathrm{m}}+\hat{\mathbf{k}}_{\rho,1})\cdot\boldsymbol{\rho}_n\right) \tag{22}$$

Note that the phase of $\widehat{H}_{\mathrm{s}\to\mathrm{meta}\to\mathrm{mr}}$ will be corrupted by the additional phase $jk_0(r_\mathrm{m} - r_\mathrm{s})$, which will cause the different phase centers for the different communication channels. Thus, this error needs to be mitigated for the multiple-channel PSK MBWC.

A phase correction term $\varphi_r = kR$ is added to the phase of the demodulated signal $\widehat{H}_{\mathrm{s}\to\mathrm{meta}\to\mathrm{mr}}(:,\mathcal{C}(t))$, where $R = r_\mathrm{m} - r_\mathrm{s}$ is the distance between the master and slavery antennas, in order to mitigate the phase error due to the difference of propagation distance between the master and slavery antennas. Corresponding experimental results are provided in **Supplementary Figure 9c,** from which the different transmitting symbols can be clearly distinguished. Finally, we perform a simple binary decision operation, and provide the decoded results in **Supplementary Figure 9d.** In addition,

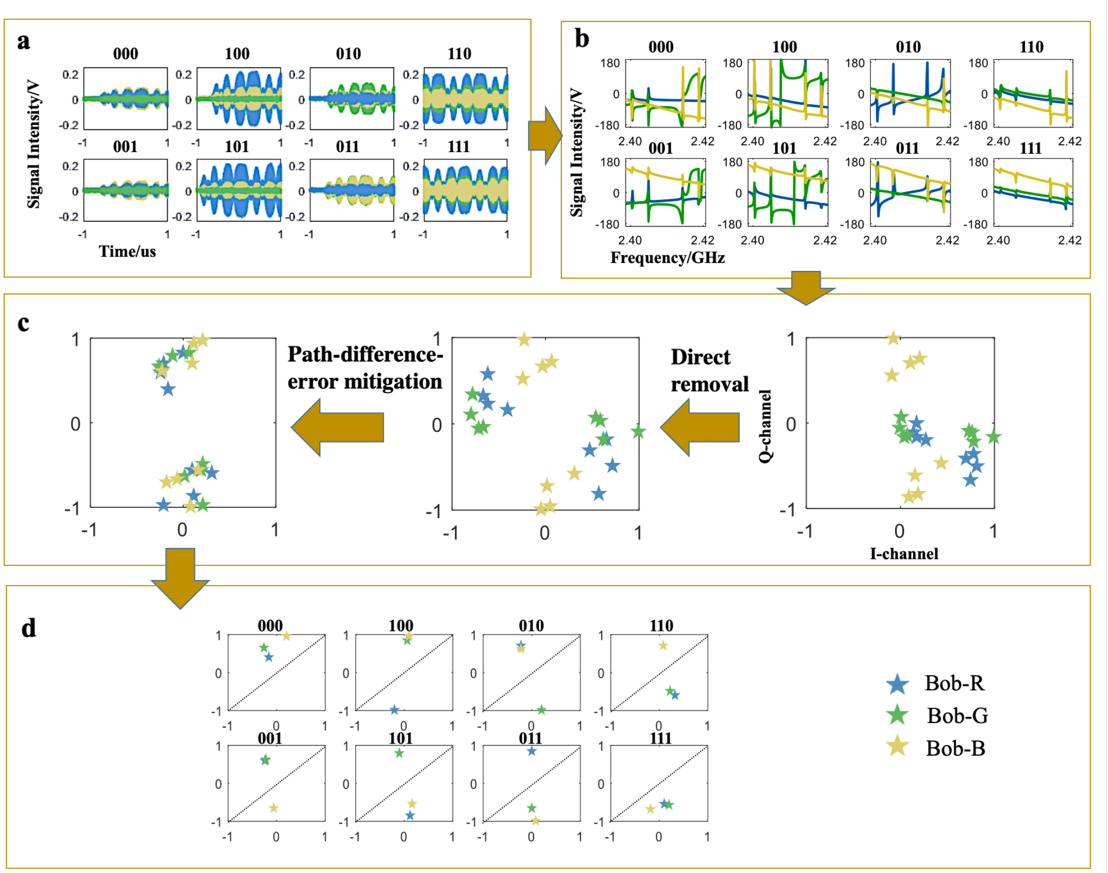

Supplementary Figure 9. Procedure of demodulation and decoding of the three-channel BPSK MBWC. **a,** The measured 2us-length Wi-Fi signals for transmitting different three-channel symbols. **b,** The phase of the demodulated signal $\widehat{H}_{\mathrm{s}\to\mathrm{meta}\to\mathrm{mr}}$ behaviors as a function of the fast-time frequency

for transmitting different three-channel symbols. **c,** the phase correction of $\widehat{H}_{\mathrm{s}\rightarrow\mathrm{meta}\rightarrow\mathrm{mr}}$ including the direct removal and phase-difference-error mitigation, **d,** the retrieved three-channel digital information.

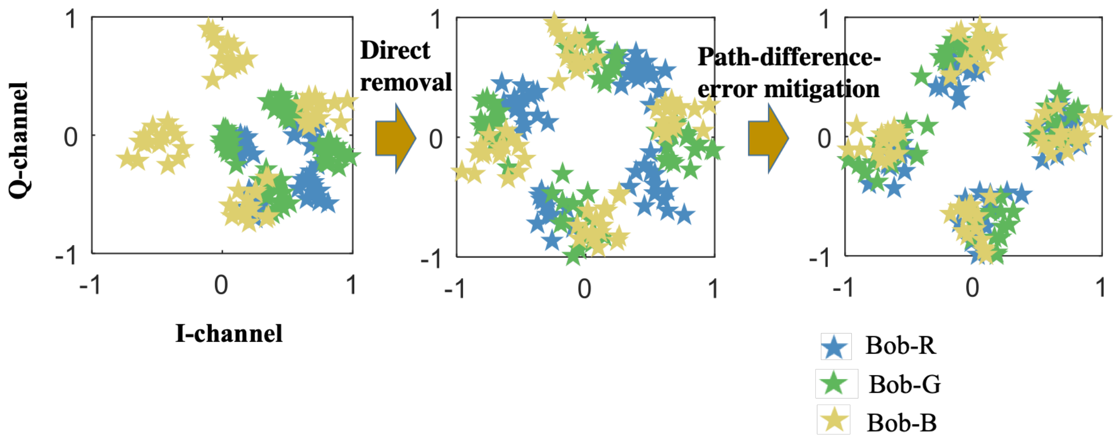

Supplementary Figure 10. Demodulation of three-channel BPSK MBWC for transmitting the color-scale image used in the second line of Supplementary Figure 5b from Alice to Bobs.

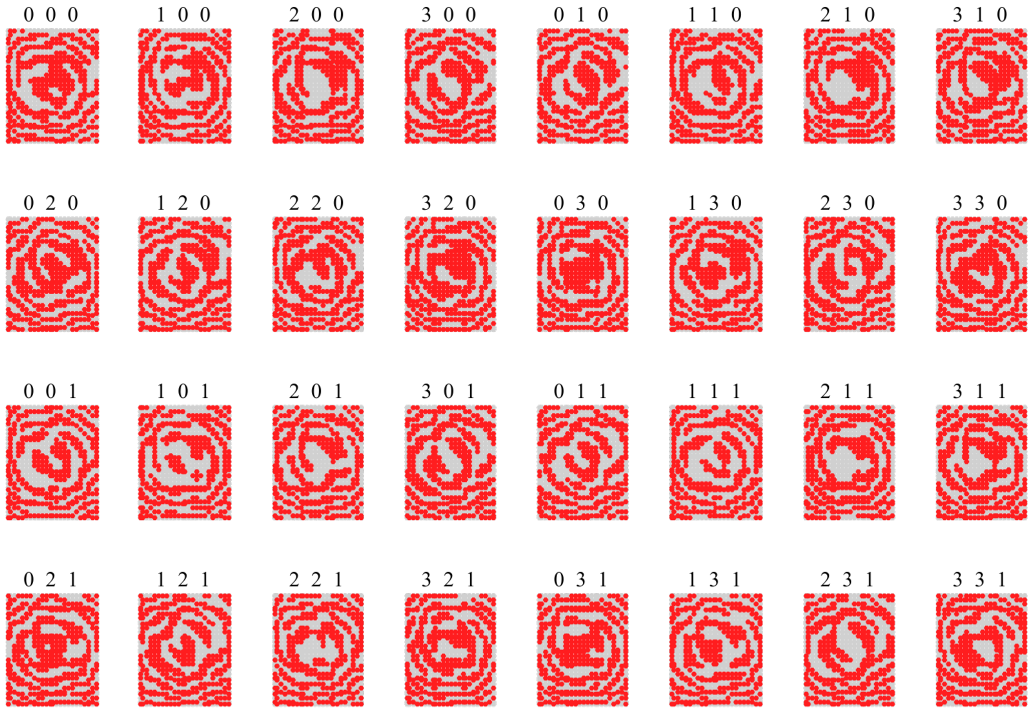

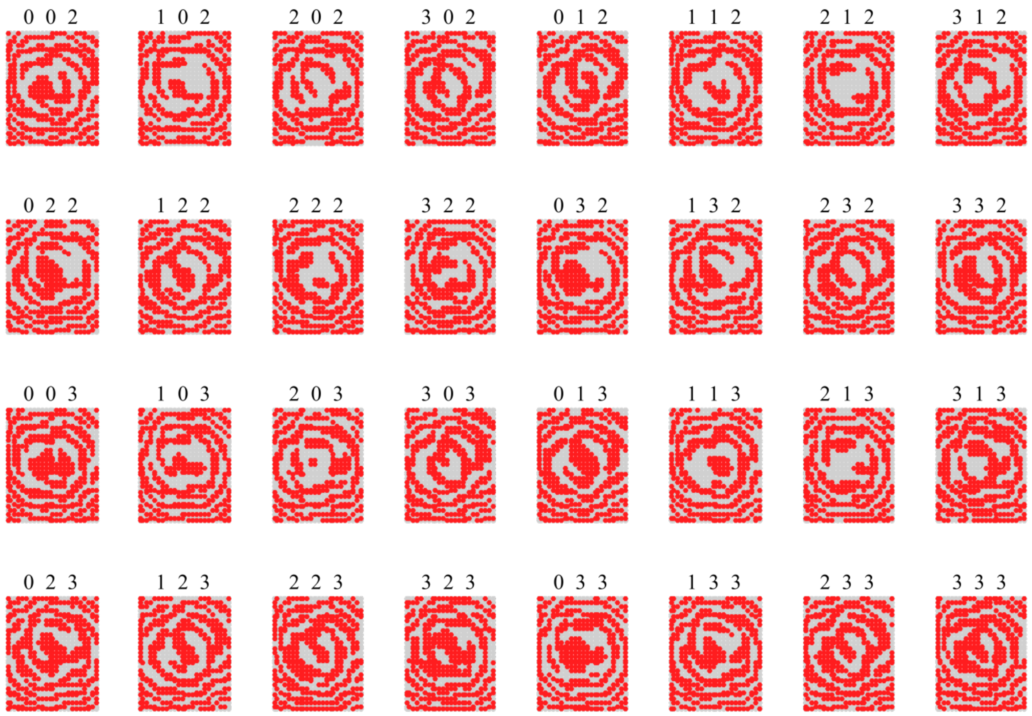

Supplementary Figure 11. Optimized 64 coding patterns for the three-channel QPSK MBWC, which are used in Figure 5 in main text.

In addition, the optimized coding patterns for the three-channel QPSK MBWC are provided in **Supplementary Figure 11**.

Supplementary Note 8. Some details about the designed MBWC demo system

To demonstrate the wide-applicability to all wave types and ease-implementation of our strategy, we build a MBWC demo under scenario of commodity 2.4GHz Wi-Fi waves. As illustrated in **Supplementary Figure 12a**, Candy transfers his information to Davy via the standard Wi-Fi active wireless communication scheme. In our demo system, Davy is downloading a zip.file from Candy. Meanwhile, Alice encodes her information into the programmable metasurface through a FPGA along with a commercial software-defined radio device (Ettus USRP X310). Here, this USRP on Alice side is used to detect the status of the non-cooperative. In particularly, if the USRP (USRP-1) detects that a Wi-Fi router

works normally, then it sends out a command to the FPGA for sending the information to the metasurface; otherwise let the FPGA to pause the information transfer. When the information-carrying metasurface is illuminated by the ambient stray Wi-Fi signals, Alice's bit stream is passively modulated by the Wi-Fi signals, and then sneakily transferred to Bob. On Bob side, two coherent receivers are set to acquire the Wi-Fi signals modulated by Alice's digital information. In addition, another USRP X310 (USRP-1) is placed on Bob side for acquiring stray Wi-Fi signals modulated by Alice's bit stream.

Some key parameters about our demo have been reported in **Figure 4**, and the frame structure of the MBWC is provided in **Supplementary Figure 12b**. Note that the data frame is controlled by a USRP on Alice side. Then, we use the MBWC demo system to demonstrate the transmission of a color-scaled video from Alice to Bob. Corresponding experimental results have been recorded in **Supplementary Video 1**. In this experiment, the MBWC BPSK is used. This video consists of three parts. The first part is for the detailed experimental setup. The second part is to show the MBWC information transmission of a color-scale video from Alice to Bob in a frame-by-frame manner, where each frame has the size of 81600 bits. The third part is to show the information transfer of the continuous video from Alice to Bob.

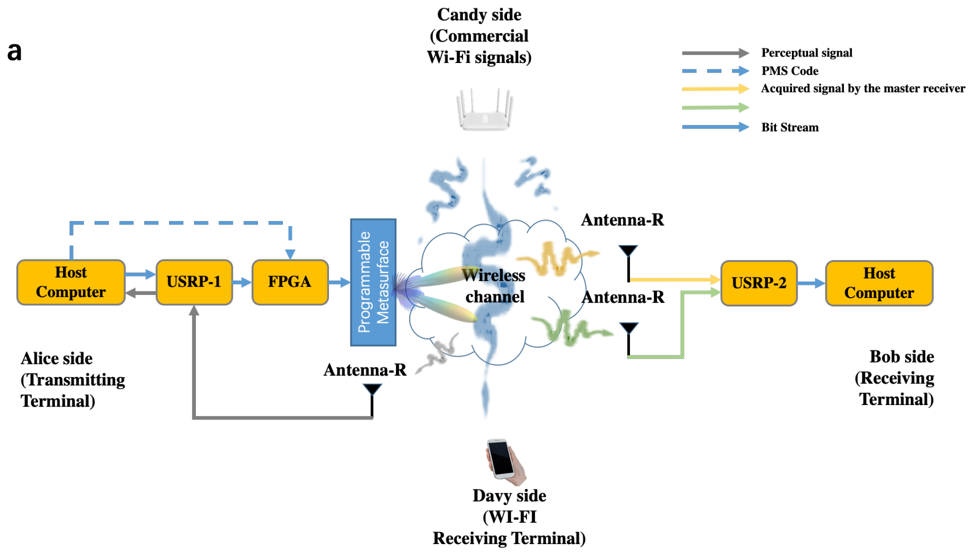

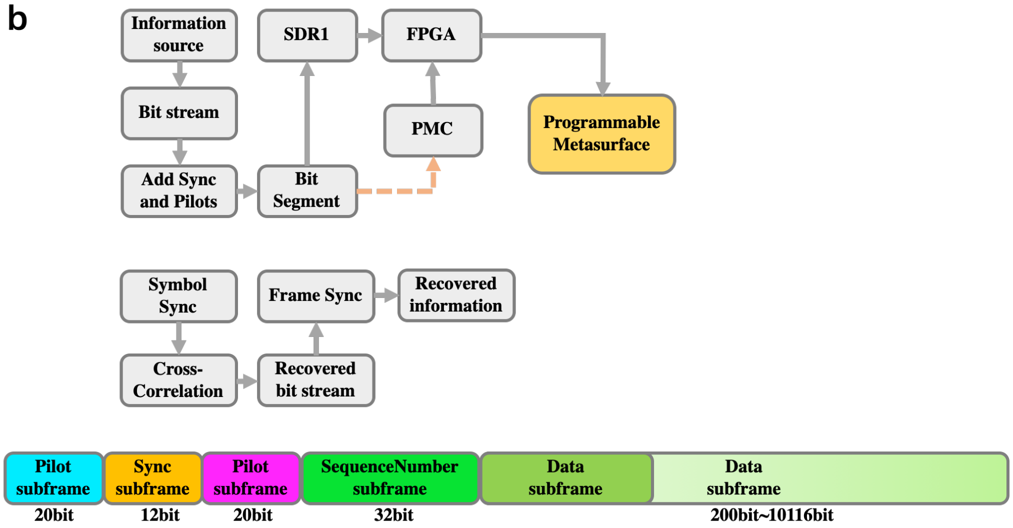

Supplementary Figure 12. a, the sketched map of the established MBWC demo. Note that a receiving antenna connected to USRP-1 is introduced on Alice side for detecting the working status of a non-cooperative Wi-Fi router. If the USRP-1detects that the router works, then it sends out a command to FPGA for starting the information transmission; otherwise pausing the information transmission. **b**, the flow of data processing and the frame structure of the proposed MBWC. In addition, corresponding results have been recorded in Supplementary Video 1.

Supplementary Video 1. Experimental results based on our demo MBWC.

Supplementary References

1. del Hougne, P., Fink, M. & Lerosey, G. Shaping Microwave Fields Using Nonlinear Unsolicited Feedback: Application to Enhance Energy Harvesting. *Phys. Rev. Applied* **8**, 061001 (2017).
2. Li, H.-Y. *et al.* Intelligent Electromagnetic Sensing with Learnable Data Acquisition and Processing. *Patterns* **1**, 100006 (2020).
