# Supplementary material for: Metasurface-assisted massive backscatter wireless communication with commodity Wi-Fi signals
Source: Nat Commun. 2020 Aug 6;11:3926. doi: 10.1038/s41467-020-17808-y (PMC7413398; doi:10.1038/s41467-020-17808-y)
Supplement: Supplementary file 3 — Description of Additional Supplementary Files [file 41467_2020_17808_MOESM3_ESM.pdf]

Description of Additional Supplementary Files

File name: Supplementary Movie 1
Description: Experimental results of our MBWC demo system
